# Data Analytics in Healthcare: A Tertiary Study

**DOI:** 10.1007/s42979-022-01507-0

**Published:** 2022-12-09

**Authors:** Toni Taipalus, Ville Isomöttönen, Hanna Erkkilä, Sami Äyrämö

**Affiliations:** Faculty of Information Technology, University of Jyväskylä, P.O. Box 35, FI-40014 Jyvaskyla, Finland

**Keywords:** Data analytics, Healthcare, Review, Machine learning, Data mining, Artificial intelligence

## Abstract

The field of healthcare has seen a rapid increase in the applications of data analytics during the last decades. By utilizing different data analytic solutions, healthcare areas such as medical image analysis, disease recognition, outbreak monitoring, and clinical decision support have been automated to various degrees. Consequently, the intersection of healthcare and data analytics has received scientific attention to the point of numerous secondary studies. We analyze studies on healthcare data analytics, and provide a wide overview of the subject. This is a tertiary study, i.e., a systematic review of systematic reviews. We identified 45 systematic secondary studies on data analytics applications in different healthcare sectors, including diagnosis and disease profiling, diabetes, Alzheimer’s disease, and sepsis. Machine learning and data mining were the most widely used data analytics techniques in healthcare applications, with a rising trend in popularity. Healthcare data analytics studies often utilize four popular databases in their primary study search, typically select 25–100 primary studies, and the use of research guidelines such as PRISMA is growing. The results may help both data analytics and healthcare researchers towards relevant and timely literature reviews and systematic mappings, and consequently, towards respective empirical studies. In addition, the meta-analysis presents a high-level perspective on prominent data analytics applications in healthcare, indicating the most popular topics in the intersection of data analytics and healthcare, and provides a big picture on a topic that has seen dozens of secondary studies in the last 2 decades.

## Introduction

The purpose of data analytics in healthcare is to find new insights in data, at least partially automate tasks such as diagnosing, and to facilitate clinical decision-making [1, 2]. Higher hardware cost-efficiency and the popularization and advancement of data analysis techniques have led to data analytics gaining increasing scholarly and practical footing in the healthcare sector in recent decades [3]. Some data analytics solutions have also been demonstrated to surpass human effort [4]. As healthcare data is often characterized as diverse and plentiful, especially big data analysis techniques, prospects, and challenges have been discussed in scientific literature [5]. Other related concepts such as data mining, machine learning, and artificial intelligence have also been used either as buzzwords to promote data analytics applications or as genuine novel innovations or combinations of previously tested solutions.

The terms *big data*, *big data analytics*, and *data analytics* are often used interchangeably, which makes the search for related scientific works difficult. Especially, *big data* is often used as a synonym for *analytics* [6], a view contested in multiple sources [7–9]. In addition, the term *data analytics* is wide and usually at least partly subsumes concepts such as statistical analyses, machine learning, data mining, and artificial intelligence, many of which overlap with each other as well in terms of, e.g., using similar algorithms for different purposes. Finally, it is not uncommon that scientific works that are not focused on technical details discuss concepts such as machine learning at different levels of specificity. For example, some studies consider merely high-level paradigms such as supervised on unsupervised learning, while some discuss different tasks such as classification or clustering, and others focus on specific modeling techniques such as decision trees, kernel methods, or different types of artificial neural networks. These concerns of nomenclature and terminology apply to *healthcare* as well, and we adapt the broad view of both healthcare and data analytics in this study. In other words, with *data analytics* we refer to general data analytics encompassing terms such as data mining, machine learning, and big data analytics, and with *healthcare* we refer to different fields of medicine such as oncology and cardiology, some closely related concepts such as diagnosis and disease profiling, and diseases in the broad sense of the word, including but not limited to symptoms, injuries, and infections.

Naturally, because of growing interest in the intersection of data analytics and healthcare, the scientific field has seen numerous secondary studies on the applications of different data analysis techniques to different healthcare subfields such as disease profiling, epidemiology, oncology, and mental health. As the purpose of systematic reviews and mapping studies is to summarize and synthesize literature for easier conceptualization and a higher level view [10, 11], when the number of secondary studies renders the subjective point of understanding a phenomenon on a high level arduous, a tertiary study is arguably warranted. In fact, we deemed the number of secondary studies high enough to conduct a tertiary study. In this study, we review systematic secondary studies on healthcare data analytics during 2000–2021, with the research goals to map publication fora, publication years, numbers of primary studies utilized, scientific databases utilized, healthcare subfields, data analytics subfields, and the intersection of healthcare and data analytics. The results indicate that the number of secondary studies is rising steadily, that data analytics is widely applied in a myriad of healthcare subfields, and that machine learning techniques are the most widely utilized data analytics subfield in healthcare. The relatively high number of secondary studies appears to be the consequence of over 6800 primary studies utilized by the secondary studies included in our review. Our results present a high-level overview of healthcare data analytics: specific and general data analytics and healthcare subfields and the intersection thereof, publication trends, as well as synthesis on the challenges and opportunities of healthcare data analytics presented by the secondary studies.

The rest of the study is structured as follows. In the next section, we describe the systematic method behind secondary study search and selection. In Section “Results” we present the results of this tertiary study, and in Section “Discussion” discuss the practical implications of the results as well as threats to validity. Section “Conclusion” concludes the study.

## Methods

### Search Strategy

**Fig. 1:**
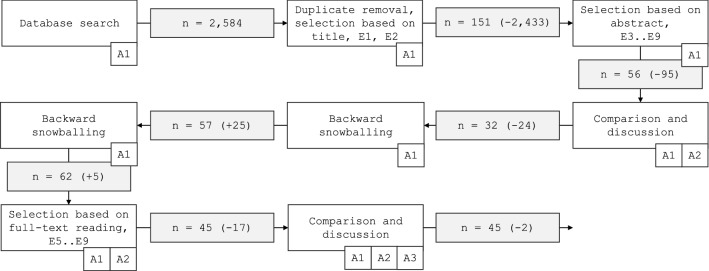
Study selection process showing the process step by step as well as the numbers of secondary studies in each step—A1, A2 and A3 refer to the authors responsible for each step, *E* refers to an exclusion criterion described in Table 2, and *n* indicates the number of included papers after a step was completed

We searched for eligible secondary studies using five databases: ACM Digital Library (ACM DL), IEEExplore, ScienceDirect, Scopus, and PubMed. In addition, we utilized Google Scholar, but the search returned too many results to be considered in a feasible timeframe. The search strings and publications returned from the respective databases are detailed in Table 1. Because the relevant terms *healthcare*, *big data* and *data analytics* have been used in an ambiguous manner in the literature, we performed two rounds of backward snowballing, i.e., followed the reference lists of included articles to capture works not found by the database searches. The search and selection processes are detailed in Fig. 1.

**Table 1:** Search strings—Scopus database search returned 16,135 results which were sorted by relevance, and the first 2,000 papers were selected for further inspection

Database	Search string	# of results
ACM DL	[Publication Title: data analy] AND [Publication Title: healthcare] AND [[Publication Title: review] OR [Publication Title: map] OR [Publication Title: systematic]] AND [Publication Date: (01/01/2000 TO 04/30/2021)]	34
IEEExplore	data analy* healthcare review	325
ScienceDirect	data analytics healthcare review; *title: healthcare; article type: review article*	118
Scopus	( TITLE-ABS-KEY ( data AND analy* AND healthcare AND review ) AND PUBYEAR> 1999 ) AND ( review )	2000*
PubMed	(healthcare[Title/Abstract]) AND (data analy*[Title/Abstract]); *limited to “systematic review”*	107

### Study Selection

After the secondary studies were searched for closer eligibility inspection, the first author applied the exclusion criteria listed in Table 2. In case the first author was unsure about a study, the second author was consulted. In case a consensus was not reached, the third author was consulted with the final decision on whether to include or exclude the study. Regarding exclusion criterion E5, we only considered secondary studies, i.e., mapping studies and different types of literature reviews. Furthermore, due to different levels of systematic approaches, we deemed a study *systematic* if (i) the utilized databases were explicitly stated (i.e., stated with more detail than “we used databases such as...” or “we mainly used Scopus”), (ii) search terms were explicitly stated, and (iii) inclusion or exclusion criteria or both were explicitly stated. Regarding exclusion criteria E6, E7 and E8, several studies considered healthcare in related fields such as healthcare from administrative perspectives [12], healthcare data privacy [13, 14], data quality [15], and comparing human performance with data analytics solutions [4]. Such studies were excluded. Similarly, studies returned by the database searches on data analytics related fields such as big data and its challenges [16], Internet-of-Things [17], and studies with a focus on software or hardware architectures behind analytics platforms [18, 19] rather than on the process of analysis were also excluded.

It is worth noting that we followed the respective secondary study authors’ classification of techniques, e.g., whether a technique is considered machine learning or deep learning. In the case a study considered more than one data analytics or healthcare subfield, we categorized the study according to what was to our understanding the primary focus. This is the reason we have refrained from defining terms such as deep learning in this study—the definitions are numerous and by defining the terms, we might give the reader the impression that we have judged whether a secondary study is concerned with, e.g., machine learning or deep learning.

**Table 2:** Exclusion (E) criteria

ID	Criterion for exclusion	Example studies excluded
E1	Published online outside the time frame 2000 to April 2021	
E2	Published in a non-peer-reviewed forum	
E3	Is not written in English	
E4	Full text we could not find or download	[20–23]
E5	Is not a systematic secondary study	[24–31]
E6	focus on data analytics but not on healthcare	[17, 18, 32]
E7	Focus on healthcare but not on data analytics	[33–35]
E8	Focus on healthcare-related field but not on healthcare	[36–39]
E9	Automatic or semi-automatic mapping	[40]

## Results

### Publication Fora and Years

**Fig. 2:**
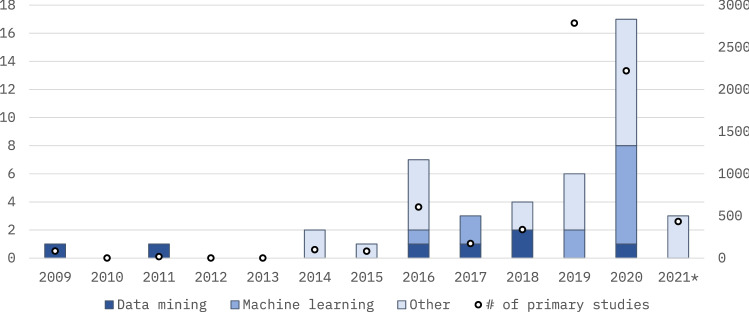
Number of included secondary studies by publication year (bars, left y-axis), and the number of included primary studies by publication year (dots, right y-axis)—the year 2021 was only considered from January to April; the figure shows that the number of secondary studies is rising

We included 45 secondary studies (abbreviated *SE* in the figures, cf. 7 for full bibliographic details). A total of 34 (76%) of the selected secondary studies were published in academic journals, nine (20%) in conference proceedings, and two (4%) were book chapters. Most of the studies were published in distinct fora (cf. Table 3), and fora with more than one selected secondary study consisted of *Journal of Medical Systems*, *International Journal of Medical Informatics*, *Journal of Biomedical Informatics*, and *IEEE Access*. As expected, the publication fora were aimed at either computer science, healthcare, or both. Finally, as can be observed in Fig. 2, the trend of systematic secondary studies in the intersection of data analytics and healthcare is growing.

**Table 3:** Publication fora

Forum	Number of studies
Journal of Medical Systems	4
International Journal of Medical Informatics	3
Journal of Biomedical Informatics	3
IEEE Access	2
Americas Conference on Information Systems (AMCIS)	1
Annals of Operations Research	1
Applied Clinical Informatics	1
Archives of Computational Methods in Engineering	1
Artificial Intelligence in Medicine	1
Australasian Conference on Information Systems	1
Biomedical Informatics Insights	1
BMC Family Practice	1
BMC Medical Informatics and Decision Making	1
Clinical Microbiology and Infection	1
Communications in Computer and Information Science	1
Computational and Structural Biotechnology Journal	1
Enterprise Information Systems	1
Healthcare	1
IEEE Computers, Software, and Applications Conference (COMPSAC)	1
IEEE International Conference on Humanoid, Nanotechnology, Information Technology, Communication and Control, Environment, and Management (HNICEM)	1
IEEE International Conference on Information Communication and Management (ICICM)	1
IEEE Reviews in Biomedical Engineering	1
IEEE Symposium on Industrial Electronics & Applications (ISIEA)	1
International Conference on Emerging Technologies in Computer Engineering	1
International Joint Conference on Biomedical Engineering Systems and Technologies	1
International Journal of Healthcare Management	1
Intensive Care Medicine	1
JAMIA Open	1
JMIR Medical Informatics	1
Journal of Diabetes Science and Technology	1
Journal of the Operational Research Society	1
Management Decision	1
NPJ Digital Medicine	1
Procedia Computer Science	1
Scientific Programming	1
Studies in Health Technology and Informatics	1
Yearbook of Medical Informatics	1
Total	45

### Secondary Study Qualities

**Fig. 3:**
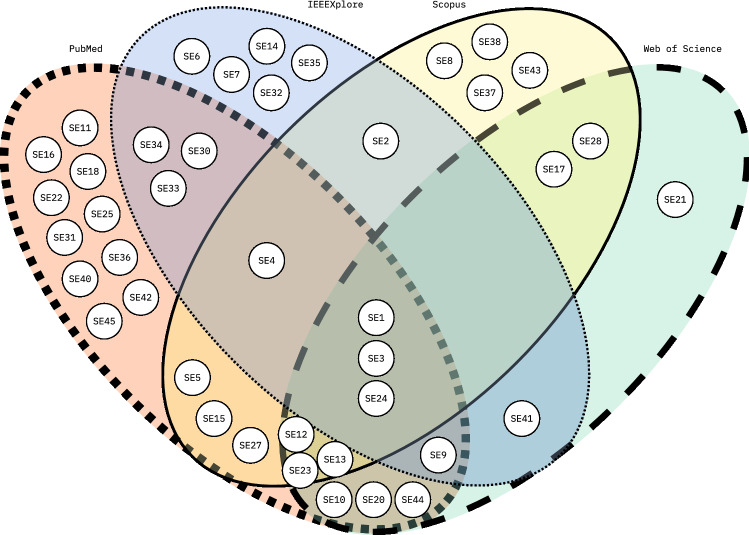
Four most popular databases used by the secondary studies were PubMed, IEEEXplore, Scopus and Web of Science—4 studies did not use any of these four databases, and other databases are not considered, e.g., the secondary study SE14, in addition to IEEExplore, might have also utilized other databases not visualized here

The selected secondary studies utilized a total of 37 different databases. The most frequently used databases were PubMed, Scopus, IEEExplore, and Web of Science, respectively. Other relatively frequently used databases were ACM Digital Library, Google Scholar, and Springer Link. Most of the secondary studies (33, or 73%) utilized four or fewer databases (*M* = 3.6, *Mdn* = 3). However, many bibliographic databases subsume others, and the number of utilized databases should not be taken as a metric for a systematic review quality. For example, a PubMed search implicitly searches MEDLINE records, and Google Scholar indexes works from most other scientific databases. The extended coverage of a wider range of academic works naturally results in numerous studies to further inspect, posing a challenge in the amount of work required. The most popular databases used in the secondary studies are visualized in Fig. 3.

**Fig. 4:**
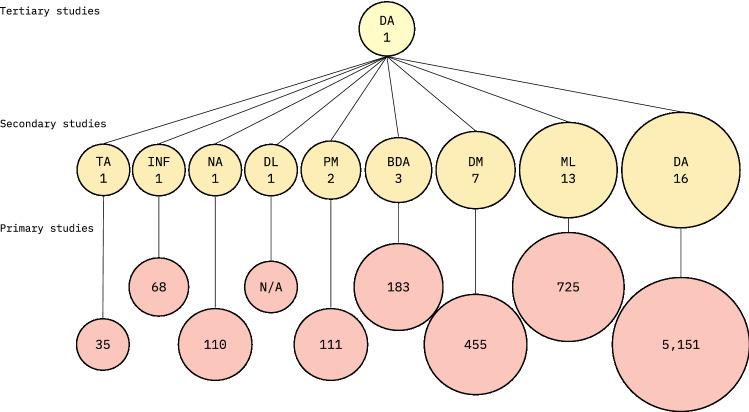
Number of secondary studies included in this tertiary study, and the number of primary studies utilized by the secondary studies, categorized by data analytics approach; *DA* general data analytics, *TA* text analytics, *INF* informatics, *NA* network analytics, *DL* deep learning, *PM* process mining, *BDA* big data analytics, *DM* data mining, *ML* machine learning; the figure shows that the general term *data analytics* was the most popular in the secondary studies

**Fig. 5:**
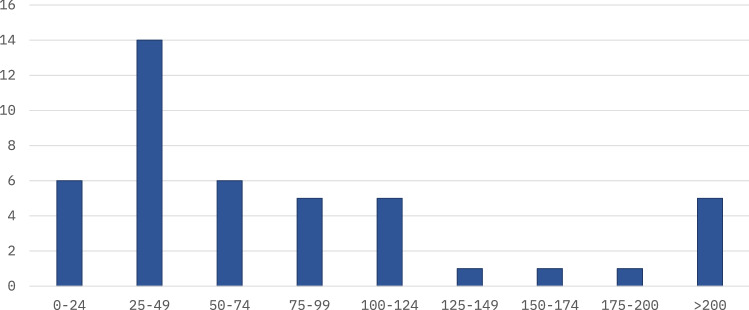
Number of primary studies (x-axis) selected for final inclusion in the secondary studies (y-axis), e.g., the chart shows that six secondary studies included 0–24 primary studies—one study (SE6) did not disclose the number of primary studies, and one study (SE15) reported two numbers: 24 primary studies for a quantitative analysis, and 28 primary studies for a qualitative analysis, and we reported that study using the latter number

The secondary studies reported an average of 155 selected primary studies (*Mdn* = 63, SD = 379.2), with a minimum of 6 (SE44) and a maximum of 2,421 primary studies (SE31). Five secondary studies selected more than 200 primary studies (cf. Fig 5). In total, the secondary studies utilized 6,838 primary studies. The number of secondary and primary studies categorized by the data analytics approach is summarized in Fig. 4.

Some secondary studies reported similar details on their respective primary studies, such as visualizations of publication years (22 studies), research approach summaries such as the number of qualitative and quantitative studies (8 studies), research field summaries (4 studies), and details on the geographic distribution of the primary study authors (5 studies). The use of PRISMA (preferred reporting items for systematic reviews and meta-analyses) [41] guidelines was reported in 15 studies.

### Subject Areas Identified

**Fig. 6:**
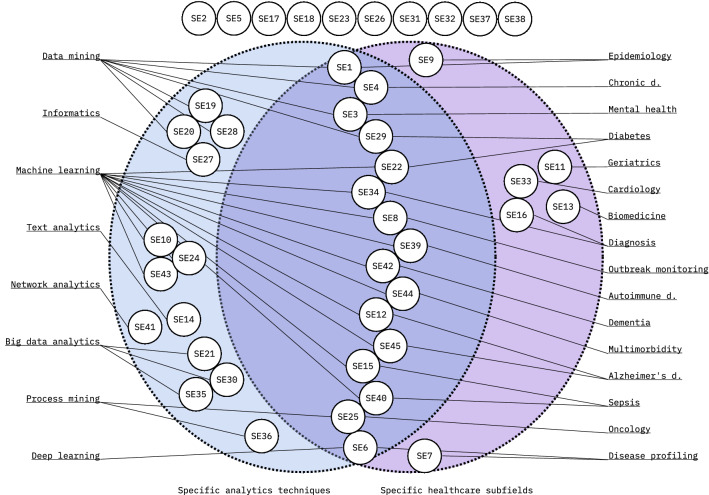
Selected secondary studies and whether they consider only specific data analytics techniques (left side), only specific healthcare subfields (right side), both (center), or neither (top); the figure may be utilized in finding relevant secondary studies on desired subfields

Some selected studies considered the relationship between healthcare in general and a specific data analysis technique, while other studies considered the relationship between data analytics in general and a specific healthcare subfield. Most of the studies, however, considered the relationship between a specific data analysis technique and a specific healthcare subfield. These considerations are summarized in Fig. 6. Readers interested mainly in general healthcare in the context of a specific analysis topic should refer to the secondary studies on the left-hand side, readers interested in general data analytics in the context of a specific healthcare topic should refer to secondary studies on the right-hand side, readers interested in a specific analysis topic applied to a specific healthcare topic should consider the studies in the middle, and readers interested in the applicability of analytics techniques in general to healthcare in general should consider the studies in the top row. Additional information on the secondary studies is presented in 6.

## Discussion

### Implications

Considering the number of primary studies utilized, only 12 studies (27%) used more than a hundred primary studies. Figure 5 seems to indicate that the threshold for conducting a literature review or a mapping study in healthcare data analytics is typically between 25 and 100 studies. Furthermore, and on the basis of the evidence currently available, it seems reasonable to argue that at least 25 primary studies (84% of the secondary studies) warrant a systematic review, and the results of systematic reviews can be seen as valuable synthesizing contributions to the field. This observation arguably also supports the relevance of this study, although this study covers a relatively large intersection of the two research areas.

The earliest included secondary study was published in 2009, which might be explained by the relative novelty of data analysis in healthcare, at least with computerized automation rather than merely applying statistical analyses. In addition, although systematic reviews are relatively common in medicine, they have only recently gained popularity and visibility in information technology [10]. As may be observed in Fig. 2, the trend of secondary studies is growing, which consequently indicates that the number of primary studies in the intersection of data analytics and healthcare is gaining research interest. The rising popularity of machine learning algorithms may be explained by the rising popularity of unstructured data, the growing utilization of graphics processing units, and the development of different machine learning tools and software libraries. Indeed, many of the techniques behind modern machine learning implementations have been around since the 1980s, but only the combination of large amounts of data, and developments in methods and computer hardware in recent years have made such implementations more cost-effective. The development of trends illustrated in, e.g., Fig. 2 propounds the view that machine learning algorithms will gain more and more practical applications in healthcare and related fields, such as molecular biology [42]. Finally, some studies have argued [43] as well as demonstrated [44, 45] that the evolution of machine learning is changing the way research hypotheses are formulated. Instead of theory-driven hypothesis formulation, machine learning can be used to facilitate the formulation of data-driven hypotheses, also in the field of medicine.

Secondary study publication fora were numerous and focused either on information technology, healthcare, or both, without obvious anomalies. The secondary studies utilized dozens of different databases in their primary study searches. It seems that the coverage of these databases is not always understood, or it is disregarded, regardless that utilizing non-overlapping databases results in less work in duplicate publication removal. For example, Scopus indexes some of ACM DL, some of Web of Science, and all of IEEExplore, effectively rendering IEEExplore search redundant if Scopus is utilized—a fact we as well understood only after conducting our searches. In addition, Google Scholar appears to index the bibliographic details of effectively all published research, yet the number of search results returned may be overwhelming for a systematic review. In practice, the selection of databases is balanced by the amount of work needed to examine the results on one end of the scales, and coverage on the other. Backward or forward snowballing may be utilized to limit the amount of work and to extend coverage.

Secondary study topics summarized in Fig. 6 give some implications for subject areas of healthcare data analytics that are mature enough to warrant a secondary study. As the figure shows, these areas are aplenty, and the most frequent data analytics techniques applied seem to be machine learning (13 secondary studies) and data mining (7 secondary studies). It is worth noting that the nomenclature we applied in this study reflects that of the secondary study authors. As explained earlier in this study, attempts at defining, e.g., machine learning and data mining in this study would inevitably contradict the definitions given in some of the included secondary studies. For further reading, Cabatuan and Maguerra [46] provide a high-level overview of machine learning and deep learning, and Shukla, Patel and Sen [47] on data mining. For more technical approaches, both Ahmad, Qamar and Rizvi [30] and Harper [48] review data mining techniques and algorithms in healthcare.

### Opportunities and Challenges in Healthcare Data Analytics

Many of the selected secondary studies provided syntheses on the current challenges and opportunities in healthcare data analytics. As the secondary studies inspected over 6800 studies of healthcare data analytics, we have summarized recurring insights here.

It was a generally accepted view in the secondary studies that healthcare data analytics is an opportunity that has already been partly realized, yet needs to be more studied and applied in more diverse contexts and in-depth scenarios [49–51]. For example, it has been noted that while big data applications are relatively mature in bio-informatics, this is not necessarily the case in other biomedical fields [52]. In general, healthcare data analytics is rather uniformly perceived as an opportunity for more cost-efficient healthcare [52, 53] through many applications such as automating a specialist’s routine tasks so that they may focus on tasks more crucial in a patient’s treatment. The cost-efficiency is likely to be more concretized by novel deep learning techniques such as large language models [54], which are also offered through implementations that perform tasks faster while consuming less resources [55]. In addition to faster diagnoses, data analytics solutions may also offer more objective diagnoses in, e.g., pathology, if the models are trained with data from multiple pathologists.

Challenges regarding healthcare data analytics are more diverse. Perhaps the most discussed challenge was the nature of the data and how it can be treated. Many secondary studies highlighted problems with missing data [56, 57], low-quality data [54], and datasets stored in various formats which are not interoperable with each other [52, 55, 56]. Furthermore, some studies raised the concern of missing techniques to visualize the outputs given by different data analyses [56, 58]. Rather intuitively, many new implementations and the increases in the amount of data require new computational infrastructure for feasible use [54, 58–60]. Some studies raised ethical concerns regarding data collection, merging, and sharing, as data privacy is a multifaceted concept [52, 54, 58, 59], especially when the datasets cover multiple countries with different legislations. Many studies also called for multidisciplinary collaboration between medical and computing experts, stating that it is crucial that the analytics implementations are based on the same vocabulary and rules as medical experts use [49, 57, 61–64], and that the technical experts understand, e.g., how feasible it is to collect training data for a model to find patterns in medical images. Closely related, many of the more complex analytics solutions operate on a *black box* principle, meaning that it is not obvious how the implementation reaches the conclusion it reaches [56, 59, 65–67]. Open solutions, on the other hand, are typically understandable only for technical experts and may be outperformed by the more complex *black box* solutions. Finally, it has been observed that the already existing analytics solutions implemented in different environments, e.g., different hospitals [56, 59, 64], are not portable into other environments. In addition, it may be that the existing solutions are not fully integrated into actual day-to-day work [57]. Fleuren et al. [68] summarize the issue aptly, urging “*to bridge the gap between bytes and bedside.*”

### Threats to Validity

As is typical for studies involving human judgment, it is possible for another group of researchers to select at least a slightly different group of studies. Furthermore, the categorization of studies into specific healthcare and data analytics topics is a likely candidate for the subject of change. We tried to mitigate the effect of human judgment by following the systematic mapping study guidelines, such as utilizing and reporting explicit exclusion criteria and search terms [11], following the PRISMA flow of information guidelines [41], and discussing discrepancies and disagreements among the authors until consensus was reached. Regarding the challenges related to the wide and rather ambiguous subject areas of data analytics and healthcare, we utilized two rounds of backward snowballing to mitigate the threat of missing relevant studies.

## Conclusion

In this study, we systematically mapped systematic secondary studies on healthcare data analytics. The results implicate that the number of secondary—and naturally primary—studies are rising, and the scientific publication fora around the topics are numerous. We also discovered that the number of primary studies included in the secondary studies varies greatly, as do the scientific databases used in primary study search. The results also show that while machine learning and data mining seem to be the most popular data analytics subfields in healthcare, specific healthcare topics are more diverse. This meta-analysis provides researchers with a high-level overview of the intersection of data analytics and healthcare, and an accessible starting point towards specific studies. What was not considered in this study is whether or not and how much the selected secondary studies overlap in their primary study selection, which could indicate the level of either deliberate or unaware overlap of similar work.

## Data Availability

The datasets generated during and/or analysed during the current study are available from the corresponding author on reasonable request.

## Appendix A. Secondary Study Qualities

See Table 4.

**Table 4:** Detailed information on the secondary studies—*PS* = number of primary studies initially considered and finally included, *PRISMA* = whether the guidelines were used, *Fields* = whether the study reports the fields of primary studies, e.g., information systems, computer science, *Years* = whether the study reports and visualizes the distribution of publication years, *Approach* = whether the study reports primary study approaches, e.g., case study, qualitative study, philosophical, *Geographic* = whether the study reports the geographic distribution of primary study authors

Study	# of PS initial	# of PS final	PRISMA	Fields	Years	Approach	Geographic
SE1	1305	8	●	○	●	○	○
SE2	73,542	81	○	●	●	●	●
SE3	211	72	○	○	○	○	○
SE4	110	32	○	○	○	○	○
SE5	16,631	32	○	●	●	●	●
SE6	*(n/a)*	69	○	○	○	○	○
SE7	*(n/a)*	*(n/a)*	○	○	○	○	○
SE8	12,648	34	○	○	○	○	○
SE9	2932	88	●	○	●	○	○
SE10	2496	35	●	○	●	○	○
SE11	696	53	●	○	○	○	○
SE12	593	37	●	○	●	○	○
SE13	9724	46	○	○	●	○	○
SE14	About 200	35	○	○	○	○	○
SE15	5281	24 (28)	●	○	○	○	○
SE16	104	12	○	○	○	○	○
SE17	6,817	804	●	●	●	●	●
SE18	761	20	○	○	○	○	○
SE19	*(n/a)*	84	○	○	○	○	○
SE20	6084	117	●	○	●	●	○
SE21	652	91	○	○	●	●	●
SE22	1287	103	○	○	●	○	○
SE23	393	41	○	○	●	●	○
SE24	270	118	●	○	●	○	○
SE25	758	37	○	○	●	○	○
SE26	*(n/a)*	321	○	●	●	●	○
SE27	755	68	○	○	○	○	○
SE28	1065	22	○	○	●	○	○
SE29	31	17	○	○	○	○	○
SE30	12,390	58	○	○	●	●	○
SE31	202,192	2,421	●	○	○	○	○
SE32	20,726	127	○	○	●	○	○
SE33	21,230	190	○	○	●	○	○
SE34	126	75	○	○	○	○	○
SE35	8355	34	○	○	●	○	○
SE36	2093	74	○	○	○	○	○
SE37	8529	576	●	○	●	○	○
SE38	5069	288	●	○	●	○	●
SE39	702	169	●	○	○	○	○
SE40	148	31	●	○	○	○	○
SE41	3695	110	○	○	○	○	○
SE42	927	35	○	○	○	○	○
SE43	2,160	101	○	○	○	○	○
SE44	2392	6	●	○	○	○	○
SE45	141	38	○	○	○	○	○

## Appendix B. Secondary Studies

1. ] Albahri AS, Hamid RA, Alwan Jk, Al-qays ZT, Zaidan AA, Zaidan BB, Albahri AOS, AlAmoodi AH, Khlaf JM, Almahdi EM, Thabet E, Hadi SM, Mohammed KI, Alsalem MA, Al-Obaidi JR, Madhloom HT. Role of biological data mining and machine learning techniques in detecting and diagnosing the novel coronavirus (COVID-19): a systematic review. J Med Syst. 2020;44(7).
2. ] Alkhatib M, Talaei-Khoei A, Ghapanchi A. Analysis of research in healthcare data analytics. In: Australasian Conference on Information Systems, 2016.
3. ] Alonso SG, de la Torre-Díez I, Hamrioui S, López-Coronado M, Barreno DC, Nozaleda LM, Franco M. Data mining algorithms and techniques in mental health: a systematic review. J Med Syst. 2018;42(9):1–15.
4. ] Alonso SG, de la Torre Diez I, Rodrigues JJPC, Hamrioui S, Lopez-Coronado M. A systematic review of techniques and sources of big data in the healthcare sector. J Med Syst. 2017;41(11):1–9.
5. ] Behera RK, Bala PK, Dhir A. The emerging role of cognitive computing in healthcare: a systematic literature review. Int J Med Inf. 2019;129:154–166.
6. ] Buettner R, Bilo M, Bay N, Zubac T. A systematic literature review of medical image analysis using deep learning. In: 2020 IEEE Symposium on Industrial Electronics & Applications (ISIEA). IEEE, 2020.
7. ] Buettner R, Klenk F, Ebert M. A systematic literature review of machine learning-based disease profiling and personalized treatment. In: 2020 IEEE 44th Annual Computers, Software, and Applications Conference (COMPSAC). IEEE, 2020.
8. ] Cabatuan M, Manguerra M. Machine learning for disease surveillance or outbreak monitoring: a review. In: 2020 IEEE 12th International Conference on Humanoid, Nanotechnology, Information Technology, Communication and Control, Environment, and Management (HNICEM). IEEE, 2020.
9. ] Carroll LN, Au AP, Detwiler LT, Fu Tc, Painter IS, Abernethy NF. Visualization and analytics tools for infectious disease epidemiology: a systematic review. J Biomed Inf. 2014;51:287–298.
10. ] Choudhury A, Asan O. Role of artificial intelligence in patient safety outcomes: systematic literature review. JMIR Med Inf. 2020;8(7):e18599.
11. ] Choudhury A, Renjilian E, Asan O. Use of machine learning in geriatric clinical care for chronic diseases: a systematic literature review. JAMIA Open. 2020;3(3):459–471.
12. ] Dallora AL, Eivazzadeh S, Mendes E, Berglund J, Anderberg P. Prognosis of dementia employing machine learning and microsimulation techniques: a systematic literature review. Proc Comput Sci. 2016;100:480–8.
13. ] de la Torre Díez I, Cosgaya HM, Garcia-Zapirain B, López-Coronado M. Big data in health: a literature review from the year 2005. J Med Syst. 2016;40(9).
14. ] Elbattah M, Arnaud E, Gignon M, Dequen G. The role of text analytics in healthcare: a review of recent developments and applications. In: Proceedings of the 14th International Joint Conference on Biomedical Engineering Systems and Technologies. SCITEPRESS—Science and Technology Publications, 2021.
15. ] Fleuren LM, Klausch TLT, Zwager CL, Schoonmade LJ, Guo T, Roggeveen LF, Swart EL, Girbes ARJ, Thoral P, Ercole A, et al. Machine learning for the prediction of sepsis: a systematic review and meta-analysis of diagnostic test accuracy. Intensive Care Med. 2020;46(3):383–400.
16. ] Gaitanou P, Garoufallou E, Balatsoukas P. The effectiveness of big data in health care: A systematic review. In: Communications in Computer and Information Science, pp. 141–153. Springer International Publishing; 2014.
17. ] Galetsi P, Katsaliaki K. A review of the literature on big data analytics in healthcare. J Oper Res Soc. 2020;71(10):1511–1529.
18. ] Gesicho MB, Babic A. Analysis of usage of indicators by leveraging health data warehouses: A literature review. In: Studies in Health Technology and Informatics, pages 184–187. IOS Press; 2019.
19. ] Iavindrasana J, Cohen G, Depeursinge A, Müller H, Meyer R, Geissbuhler A. Clinical data mining: a review. Yearb Med Inf. 2009;18(01):121–133.
20. ] Islam Md, Hasan Md, Wang X, Germack H, Noor-E-Alam Md. A systematic review on healthcare analytics: Application and theoretical perspective of data mining. Healthcare. 2018;6(2):54.
21. ] Kamble SS, Gunasekaran A, Goswami M, Manda J. A systematic perspective on the applications of big data analytics in healthcare management. Int J Healthc Manag. 2018;12(3):226–240.
22. ] Kavakiotis I, Tsave O, Salifoglou A, Maglaveras N, Vlahavas I, Chouvarda I. Machine learning and data mining methods in diabetes research. Comput Struct Biotechnol J. 2017;15:104–116.
23. ] Khanra S, Dhir A, Najmul Islam AKM, Mäntymäki M. Big data analytics in healthcare: a systematic literature review. Enterp Inf Syst. 2020;14(7):878–912.
24. ] Sudheer Kumar E, Shoba Bindu C. Medical image analysis using deep learning: a systematic literature review. In: International Conference on Emerging Technologies in Computer Engineering, pages 81–97. Springer; 2019.
25. ] Kurniati AP, Johnson O, Hogg D, Hall G. Process mining in oncology: a literature review. In: 2016 6th International Conference on Information Communication and Management (ICICM). IEEE, 2016.
26. ] Li J, Ding W, Cheng H, Chen P, Di D, Huang W. A comprehensive literature review on big data in healthcare. In: Twenty-second Americas Conference on Information Systems (AMCIS), 2016.
27. ] Luo J, Wu M, Gopukumar D, Zhao Y. Big data application in biomedical research and health care: A literature review. Biomed Inf Insights. 2016;8:BII.S31559.
28. ] Malik MM, Abdallah S, Ala’raj M. Data mining and predictive analytics applications for the delivery of healthcare services: a systematic literature review. Ann Oper Res. 2016;270(1-2):287–312.
29. ] Marinov M, Mohammad Mosa AS, Yoo I, Boren SA. Data-mining technologies for diabetes: A systematic review. J Diabetes Sci Technol. 2011;5(6):1549–1556.
30. ] Mehta N, Pandit A. Concurrence of big data analytics and healthcare: a systematic review. Int J Med Inf. 2018;114:57–65.
31. ] Mehta N, Pandit A, Shukla S. Transforming healthcare with big data analytics and artificial intelligence: a systematic mapping study. J Biomed Inf. 2019;100:103311.
32. ] Nazir S, Khan S, Khan HU, Ali S, Garcia-Magarino I, Atan RB, Nawaz M. A comprehensive analysis of healthcare big data management, analytics and scientific programming. IEEE Access. 2020;8:95714–95733.
33. ] Nazir S, Nawaz M, Adnan A, Shahzad S, Asadi S. Big data features, applications, and analytics in cardiology—a systematic literature review. IEEE Access. 2019;7:143742–143771.
34. ] Peiffer-Smadja N, Rawson TM, Ahmad R, Buchard A, Georgiou P, Lescure F-X, Birgand G, Holmes AH. Machine learning for clinical decision support in infectious diseases: a narrative review of current applications. Clin Microbiol Infect. 2020;26(5):584–595.
35. ] Raja R, Mukherjee I, Sarkar BK. A systematic review of healthcare big data. Sci Programm. 2020;2020.
36. ] Rojas E, Munoz-Gama J, Sepúlveda M, Capurro D. Process mining in healthcare: a literature review. J Biomed Inform. 2016;61:224–236.
37. ] Salazar-Reyna R, Gonzalez-Aleu F, Granda-Gutierrez EMA, Diaz-Ramirez J, Garza-Reyes JA, Kumar A. A systematic literature review of data science, data analytics and machine learning applied to healthcare engineering systems. Manag Decis. 2020.
38. ] Secinaro S, Calandra D, Secinaro A, Muthurangu V, Biancone P. The role of artificial intelligence in healthcare: a structured literature review. BMC Med Inf Decis Making. 2021;21(1).
39. ] Stafford IS, Kellermann M, Mossotto E, Beattie RM, MacArthur BD, Ennis S. A systematic review of the applications of artificial intelligence and machine learning in autoimmune diseases. NPJ Digit Med. 2020;3(1):1–11.
40. ] Teng AK, Wilcox AB. A review of predictive analytics solutions for sepsis patients. Appl Clin Inf. 2020;11(03):387–398.
41. ] Toor R, Chana I. Network analysis as a computational technique and its benefaction for predictive analysis of healthcare data: a systematic review. Arch Comput Methods Eng. 2020;28(3):1689–1711.
42. ] Tsang G, Xie X, Zhou S-M. Harnessing the power of machine learning in dementia informatics research: Issues, opportunities, and challenges. Rev Biomed Eng. 2020;13:113–129.
43. ] Waring J, Lindvall C, Umeton R. Automated machine learning: review of the state-of-the-art and opportunities for healthcare. Artif Intell Med. 2020;104:101822.
44. ] Waschkau A, Wilfling D, Steinhäuser J. Are big data analytics helpful in caring for multimorbid patients in general practice?—a scoping review. Family Pract. 2016;20(1).
45. ] Zhang R, Simon G, Yu F. Advancing Alzheimer’s research: a review of big data promises. Int J Med Inf. 2017;106:48–56.
